# Gain a Baby Lose a Tooth—Is There an Association between Periodontitis and Preterm Birth?

**DOI:** 10.3390/jcm11237183

**Published:** 2022-12-02

**Authors:** Valentin Bartha, Sahra Steinmacher, Rebekka Wittlinger, Sébastien Boutin, Jan Pauluschke-Fröhlich, Christiane von Ohle, Sara Yvonne Brucker, Thomas Bruckner, Diana Wolff

**Affiliations:** 1 Department of Restorative Dentistry, Tuebingen University Hospital, Osianderstraße 2, 72076 Tuebingen, Germany; 2 Center for Conservative Dentistry and Periodontology, Heidelberg University Hospital, Im Neuenheimer Feld 400, 69120 Heidelberg, Germany; 3 Department of Women’s Health, Tuebingen University Hospital, Calwerstr. 7, 72076 Tuebingen, Germany; 4 Center for Infectiology, Heidelberg University Hospital, Im Neuenheimer Feld 324, 69120 Heidelberg, Germany; 5 Institute for Medical Biometry/Biostatistics, Heidelberg University Hospital, Im Neuenheimer Feld 130, 69120 Heidelberg, Germany

**Keywords:** periodontitis, preterm birth, oral microbiome, 16S rRNA sequencing

## Abstract

Preterm birth serves as one of the leading causes of neonatal mortality worldwide. The underlying mechanisms that contribute to preterm birth are not yet fully understood. However, an association between periodontitis and preterm birth has been proposed. The periodontal status and presence of periodontal pathogens in women with different birth outcomes have been previously examined. However, varying definitions of periodontitis and different microbiological methods make their interpretation challenging. The aim of this case-control study on women with and without preterm birth was to investigate their periodontal status using the current classification system for periodontal diseases. Moreover, differences in the periodontal microbiome of the study participants were investigated. Therefore, we collected data on oral and periodontal parameters in 77 puerperal women divided into two groups based on gestational age at delivery: 33 patients with preterm birth (PTB, <37 weeks) and 44 patients with term birth (TB, >37 weeks). These data included pocket probing depth (PPD), clinical attachment loss (CAL), bleeding on probing (BOP), gingival-bleeding index, DMFT index, and gynecologic and dental history. In addition, their oral microbiome was explored. Median CAL and percentage PPD ≥ 4 mm were significantly higher in the PTB group than in the TB group (*p* = 0.0128 and *p* = 0.047, respectively). Birth weight was significantly higher in periodontally healthy women than in those with gingivitis (*p* = 0.0078) or periodontitis (*p* = 0.0127). The periodontal microbiome differed significantly between groups. Our results are underlining the possible association between periodontitis and preterm delivery. Women with periodontitis had babies with significantly lower birth weights. The microbiome varied between the groups.

## 1. Introduction

Preterm or premature birth is one of the leading causes of neonatal mortality worldwide [1,2]. About 10% of all births are preterm and account for up to 75% of all perinatal deaths and more than 50% of developmental disorders in children [3]. Therefore, the prevention of preterm birth would significantly impact public health resources.

Almost half of all premature births are due to preterm labor, with preceded premature rupture of membranes (PPROM) involved in 25–30%. One-third account for maternal or fetal indications [4,5]. It is assumed that PPROM is primarily caused by ascending intrauterine infection leading to decidual activation and preterm labor [6,7]. However, to what extent other systemic infections may contribute to the occurrence of PPROM remained still to be determined [4,8]. Recently, a dysbiotic vaginal microbiome with a higher diversity has been described as an important risk factor for PPROM, underlining the role of inflammatory mechanism in context of preterm birth [9].

Periodontal diseases are affecting up to 90% of the worldwide population and are discussed as a risk factor for adverse pregnancy outcomes [10]. While gingivitis only affects the marginal gums, periodontitis leads to a progredient loss of tooth-supporting connective tissue including the tooth surrounding alveolar bone structures [11]. A dysbiotic dental plaque formation in combination with modifying factors like malnutrition, western diet conditions, medication, chronic stress, smoking habits, metabolic and/or hemorrhagic diseases or specific alterations of hormone levels during adolescence or pregnancy are currently known as etiological factors for gingivitis [12]. In case of simultaneous occurrence of such mechanisms, and especially in combination with genetic factors like specific single nucleotid polymorphisms, the immunological mechanisms are getting more likely to fail in stopping the progression of inflammation on the gum level [13]. This leads to an additional connective tissue and bone loss, defined as periodontitis [14]. Simultaneous with the tissue breakdown, the dysbiotic plaque begins to grow deep into the newly arisen anatomic niche between the gum surface and the tooth surface, called periodontal pocket [10]. Within this periodontal pocket, the diversity of the dysbiotic subgingival microbiome begins to increase [15]. Specific keystone pathogens have been described to trigger the formation of this dysbiosis [16] and to subvert immunological mechanisms. These pathogens are also dispersed into the blood stream, affecting various other organ systems [17]. Current treatment strategies are mainly based on control of the dysbiotic biofilm by repeated mechanical cleaning of supra- and subgingival sites of affected teeth [18]. At the latest since the definition of the ecological plaque hypotheses [19], the question arose why a principally symbiotic dental plaque formation gets dysbiotic. Not only the occurrence of pathogenic bacteria but also significant ecological shifts like changes in pH-values, temperature, nutrients and local atmospheric shifts are necessary to alter a symbiotic bacterial community into a pathogenic dysbiotic community [19]. Regarding periodontal diseases, it is discussed if an elevated systemic inflammatory state might also establish an inflammatory environment in the gums, resulting in favorable conditions for inflammophilic bacteria [20]. Hence, recently adjunctive treatment strategies like nutraceutical and diet interventions, which might be able to modulate the systemic level of inflammation, were investigated [21].

The underlying mechanisms that lead to preterm birth are not yet fully understood. However, two pathomechanisms have been proposed that may explain the association between periodontitis and preterm birth: (1) directly through the invasion of microorganisms to the placenta and amniotic cavity via hematogenous dissemination, inducing an immune reaction within the fetal-placental unit; and (2) indirectly through inflammatory mediators produced in periodontal and feto-placental tissues as an immune response to the invasion of pathogens [2,22,23].

However, oral pathogens have only rarely been detected in intrauterine infections in patients with preterm labor [24]. Bacteria found in the placenta of patients with preterm labor are not usually seen in the lower genitourinary tract [25]. This observation might add weight to the hypothesis that elevated periodontitis-associated inflammatory mediators affect intra-uterine inflammation [25] and stimulate uterus contractility, causing PPROM [26].

Moreover, the bidirectional association of periodontitis and diabetes mellitus, including gestational diabetes, affects the risk for preterm birth [27]. Diabetes serves as one trigger mechanism for the occurrence of oxidative stress, resulting from an imbalance between reactive oxygen species (ROS) and physiological detoxification abilities [28]. ROS have been reported to be associated with many adverse pregnancy outcomes including preterm birth and fetal growth restriction [28].

Periodontitis and the related systemic dissemination of periodontal pathogenic bacteria was described as a risk factor for cardiovascular diseases (CVD) [29]. A proposed mechanism is the trigger function that periodontal inflammation seems to have on an elevated state of systemic inflammation [30]. Similarly, CVD and its known risk factors like diabetes or hypertension were reported as associated with preterm birth [31]. Hence, beside the proposed patho-mechanisms for the association between periodontitis and preterm birth, there seems to be an interconnection between various other risk factors. Hereby, periodontitis might not only affect adverse pregnancy outcomes in a direct way, but also various systemic diseases which themselves are associated with preterm birth.

Recently, a high prevalence of preterm birth and low birth weight was reported in women with periodontitis and a deficiency of 25-hydroxy-vitamin D in context with its anabolic effect on growth [32].

The periodontal status and presence of periodontal pathogens in women with different birth outcomes have previously been examined in several studies [24,33,34,35,36]. Limitations for an overall interpretation of the current evidence on this topic are a high variability of definitions of periodontitis [37] and the methodology of measurements (e.g., number of measured sites per tooth, measurement of different variables), and the use of different microbiological methods—especially the fact that only a limited number of studies used next generation sequencing methods [38]. Moreover, while some studies reported a correlation between periodontal status and preterm birth [24,33,34], others found a higher degree of gingival inflammation but no periodontitis in women with preterm birth [35,36]. Hence, following suggestions of systematic reviews on that topic, in this study [39] we investigated the oral status—including periodontal diagnosis based on the *2017 Classification of periodontal diseases* [40,41]—and systemic and gynecological clinical data, and also oral microbiome 16S rRNA sequencing data in women with preterm and term birth to clarify the association between periodontitis and preterm birth, hypothesizing that there are worse periodontal parameters in women with preterm birth and differences in the periodontal microbiome.

## 2. Materials and Methods

Puerperal patients who had given birth between July 2018 and January 2021 in the Department of Women’s Health at the University of Tuebingen were enrolled in this case-control study. Patients with preterm birth <37 weeks (PTB group) and those with term birth (TB group) were examined after delivery. Participants were comprehensively informed and provided written informed consent before participation. This study was designed as a case-control study and followed the STROBE statement (Supplementary Table S1). It was approved by the ethical committee of the University of Tuebingen (protocol number 445/2018BO) and registered in the German Clinical Trials Register (DRKS 00026202).

### 2.1. Inclusion- and Exclusion Criteria

Women of 18 years or older were eligible for participation. Patients with an intake of medication with influence on gingival proliferation, multiple pregnancies, polyhydramnios, malformation of the fetus, or placenta praevia were excluded, as were patients with severely impaired health status, such as an infectious disease or diabetes.

### 2.2. Demographic and Clinical Data 

Age at the examination, educational background, smoking habits, general diseases, and medication were assessed in PTB and TB groups. The gynecological parameters gravity and parity, delivery mode, gestational age at delivery, and birth weight were recorded. Oral examinations of these hospitalized women were performed in the Department of Women’s Health 1–4 days after delivery using a headlamp and loupe glasses. Periodontal examination comprised a full-mouth periodontal status, including probing pocket depth (PPD) and clinical attachment loss (CAL) measured at six sites per tooth. PPD was measured in mm using a manual periodontal probe (PCPUNC−15, Hu-Friedy, Chicago, USA). The percentage of bleeding sites was assessed at four sites for each tooth to evaluate the state of inflammation of the marginal gingiva and recorded as bleeding on probing (BOP) [42,43]. 

The number of teeth, missing teeth, decayed teeth and filled teeth were recorded (DMFT index) [44]. Gingival bleeding was recorded using the Gingival bleeding index (GBI) [43]. Two calibrated examiners of the Department of Conservative Dentistry and Periodontology at the University of Tuebingen examined patients. A standard calibration process was performed by measuring periodontal parameters (PPD and CAL) until an allowed variance of 1 mm was achieved [45]. The periodontal inflamed surface area (PISA) was calculated according to Nesse et al. [46]. The periodontal diagnosis was classified using the current classification [47]. In the case of periodontitis, the stage was determined based on the clinical data. Information on delivery mode, occurrence of gestational diabetes, number of previous miscarriages and socioeconomical status were collected.

### 2.3. Microbiological Analysis

Supragingival and subgingival plaque specimens were collected. Patients ate a similar diet due to their meal plan in the hospital at least two days prior to sampling. They followed their habitual oral hygiene regime without further professional instructions or advice. Samples were obtained after patients abstained from oral hygiene for at least two hours.

Local moisture control was obtained by applying cotton rolls around the sampling site. The supragingival plaque was swiped off from at least four different tooth surfaces in different mouth locations, using a sterile periodontal probe or curette and transferred to a 1.5 mL Eppendorf tube. If applicable, the samples were taken from sites with signs for inflammation.

In participants with periodontitis and probing depths ≥ 4 mm, subgingival microbiological samples were collected from the deepest pocket of at least 4 mm and bleeding on probing. After carefully removing supragingival plaque, six sterile paper points (ISO 35) were inserted into the sulcus for 10 s each and pooled in a sterile, 1.5 mL Eppendorf tube. Additionally, six sterile paper points from the same batch served as a negative control to screen for contamination and were pooled in one separate tube per patient. All samples were frozen at −25 °C until required.

### 2.4. Isolation of Bacterial DNA 

Bacterial DNA was isolated according to a published protocol and extracted using a DNeasy Blood and Tissue Kit (Qiagen, Hilden, Germany) according to the manufacturer’s protocol [48,49]. Additional enzymatic lysis was performed using lysozyme (20 mg/mL) and mutanolysin (1.500 U/mL; Sigma-Aldrich, Taufkirchen, Germany).

### 2.5. Library Preparation for Next-Generation Sequencing (NGS)

DNA was amplified using universal bacterial primers targeting the V4 region of the 16S rRNA gene (515F and 806R from [50]). Each sample was assigned a unique barcode that was attached to each primer before it was linked to the Illumina adapters. The PCR reaction mix contained Q5 High-Fidelity 1X Master Mix (New England Biolabs GmbH, Germany), 0.5 µM of each primer, 2 µL of DNA, and sterile water for a final volume of 25 µL. The thermal cycle was as follows: the first denaturation at 94 °C for 3 min, followed by 30 amplification cycles (94 °C for 45 sec, 50 °C for 1 min, and 72 °C for 1 min 30 sec), and the final extension at 72 °C for 10 min, performed in a Primus 25 (Peqlab Biotechnologie GmbH, Germany) or FlexCycler^2^ (Analytik Jena AG, Germany) PCR machine. Negative controls using sterile water as a template and mock community control (ZymoBIOMICS Microbial Community Standard; ZymoResearch, Germany) were processed in parallel to control for contamination. PCR products were evaluated via Qiaxcel DNA screening (Qiagen, Germany) and then purified using Agencourt AMPure XP beads (Beckman Coulter, Germany) according to the manufacturer’s recommended protocol. Purified products were checked for quality and concentration using the Quant-iT™ PicoGreen^®^ dsDNA Assay Kit (ThermoFisher Scientific GmbH, Dreieich, Germany). An equimolar mix of all the PCR products was sequenced in-house on an Illumina MiSeq sequencing system (2× 300 cycles; Berlin, Germany).

### 2.6. Analysis of Sequences

Processing of raw sequence reads was performed using *DADA2* [51]. They were subjected to quality control—no ambiguities (N), less than one error per read, truncation of the reads after quality score < 2—merged into contigs and checked for chimera with default parameters. Ribosomal sequence variants (RSVs) were assigned to taxonomy using the Silva database (Version 138). Because the negative control produced amplicons and was therefore sequenced, we used the package *decontam* [52] following the stringent “either” method to remove potential contaminants from our dataset. This method uses prevalence or frequency methods to call the contaminants. Each RSV considered as a contaminant was removed from the dataset. The remaining RSVs were used to calculate descriptive indices for α-diversity (Shannon index), richness (number of observed RSVs), evenness (Pielou index), and dominance (relative abundance of the most dominant RSV). The significance of differences in α-diversity indices between the two groups was assessed with the Mann–Whitney U test. The β-diversity was calculated based on the weighted Unifrac index, and statistical differences were estimated using permutational multivariate analysis of variance (PERMANOVA). The differential abundance in the taxa between groups was assessed using *Deseq2* at each taxonomic level. A generalized linear model was used to estimate the impact of the clinical data on preterm birth risk. All analyses were performed in the R statistical software (v.4.1.0).

### 2.7. Statistical Analysis of Clinical Data

Clinical data were statistically analyzed using SAS JMP (SAS-Institute GmbH, Heidelberg, Germany). The determined cohort size was 60 patients each for the case and control groups. This cohort size aimed at the estimation of the effect for a subsequent confirmatory study. Formally, assuming 20% intraindividual comparison of 0.597 standard deviations, interindividual comparison of 0.826 standard deviations can be demonstrated. Descriptive data were calculated as absolute and relative frequencies, medians, and interquartile ranges (IQRs). Because the data were non-normally distributed (Anderson–Darling Test *p* < 0.01), differences between groups were assessed using either a Mann–Whitney U- or Kruskal–Wallis test. Categorial data comparisons were performed using a Chi-square test. Spearman’s rank correlation coefficient was used to assess potential correlations between clinical variables. Following Cohen (1988) a Spearman’s ρ of 0.1 to 0.29 is considered as weak, 0.3 to 0.49 moderate, and a Spearman’s ρ over 0.49 as a strong correlation. Because the *p*-values of Spearman analysis are highly influenced by the sample size and hence, to avoid a bias in the data interpretation of the Spearman analysis, they were not reported for the data of this study.

## 3. Results

### 3.1. Pregnancy Outcome and Clinical Data

**Figure 1:**
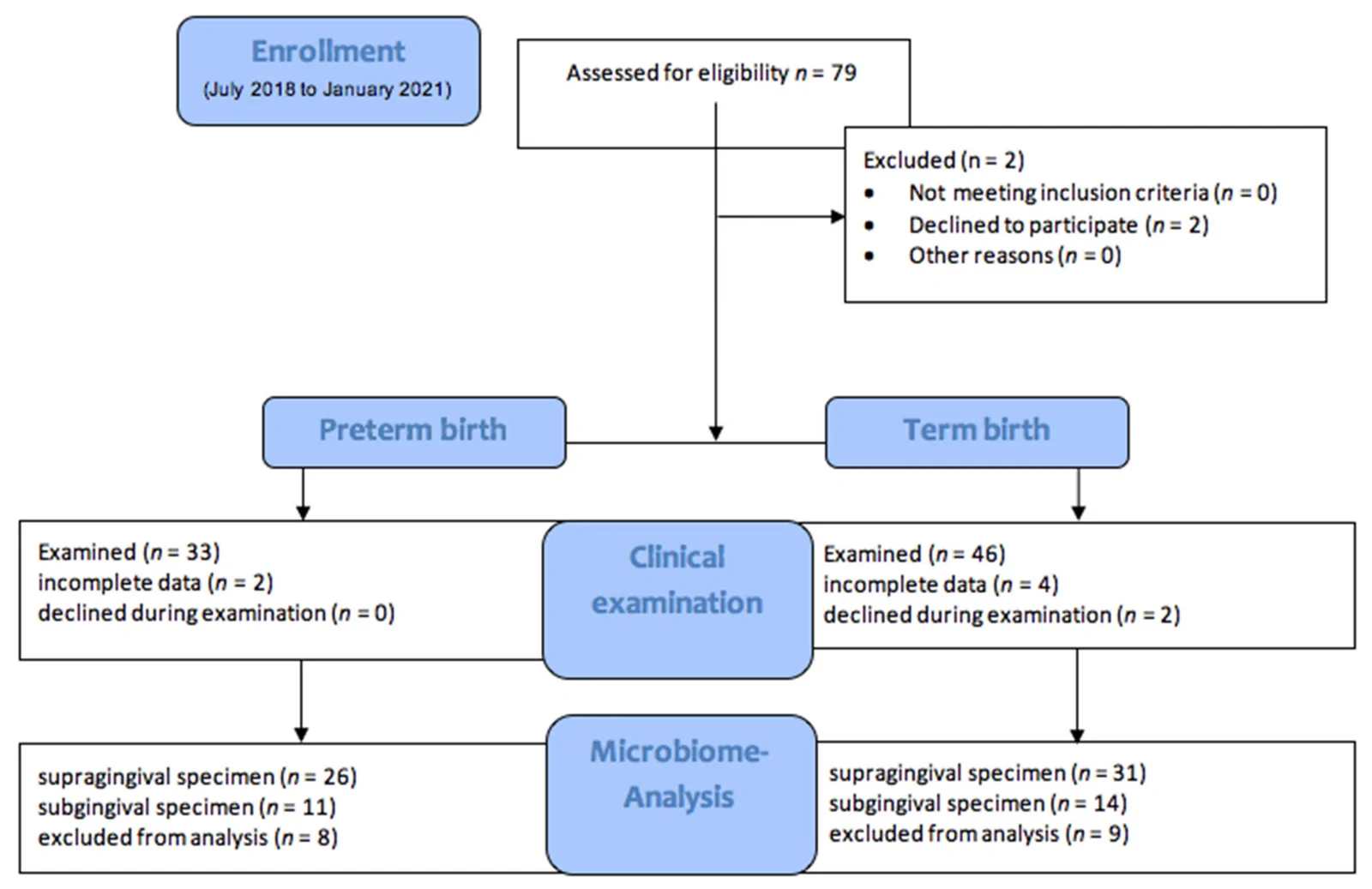
Study Flow chart.

A total of 79 women were enrolled in this study: 33 had a PTB (≤37 weeks of pregnancy), and 46 had a TB (>37 weeks). Two patients with term birth declined during the examination (Figure 1).

The median age of women in the PTB (33; min = 21 and max = 44) and TB (29.5; min = 19 and max = 41) groups did not differ significantly. The median delivery week in the PTB group was 35.14 (IQR 34.00–36.14) weeks and 39.64 (IQR 38.75–40.71) weeks in the TB group. This difference was statistically significant (*p* < 0.001). The median birth weight was significantly lower in the PTB group (2620 g; IQR 2285.00–2787.50) than in the TB group (3375 g; IQR 3090.00–3615.00; *p* < 0.001). Spontaneous birth was significantly lower in the PTB group than in the TB group (*p* = 0.001; Table 1).

Clinical examinations revealed the PTB group to have significantly higher median CAL (1.4; IQR 0.4–1.9) and percentage PPD ≥ 4 mm (2.98; IQR 1.19–5.95) than the TB group (CAL: 0.4; IQR 0.0–1.2; PPD ≥ 4 mm: 2; IQR 0–6; Intergroup *p* = 0.0128 and *p* = 0.047, respectively). While DMFT, mean PPD, and absolute number of PPD ≥ 4 mm were higher in the PTB group than in the TB group, the difference was not statistically significant. Similarly, BOP, PISA, GBI values were not significantly different between groups (Table 2). Education and socioeconomical status did not impact the observed clinical parameters (data not shown).

Converting the clinical data into periodontal diagnosis, we observed a higher percentage of women with oral health and gingivitis in the TB group than in the PTB group, while the number and percentage of women with Stage I–III periodontitis were instead higher in the PTB group (Table 3). However, none of the differences were statistically significant. Similarly, no significant differences were observed in mean age and number of smokers between different periodontal diagnosis groups (Table 3). When considering all participants, we found a weak to moderate negative correlation between birth weight and the relative number of pockets ≥ 4 mm (Spearman’s *ρ* = −0.293).

**Figure 2:**
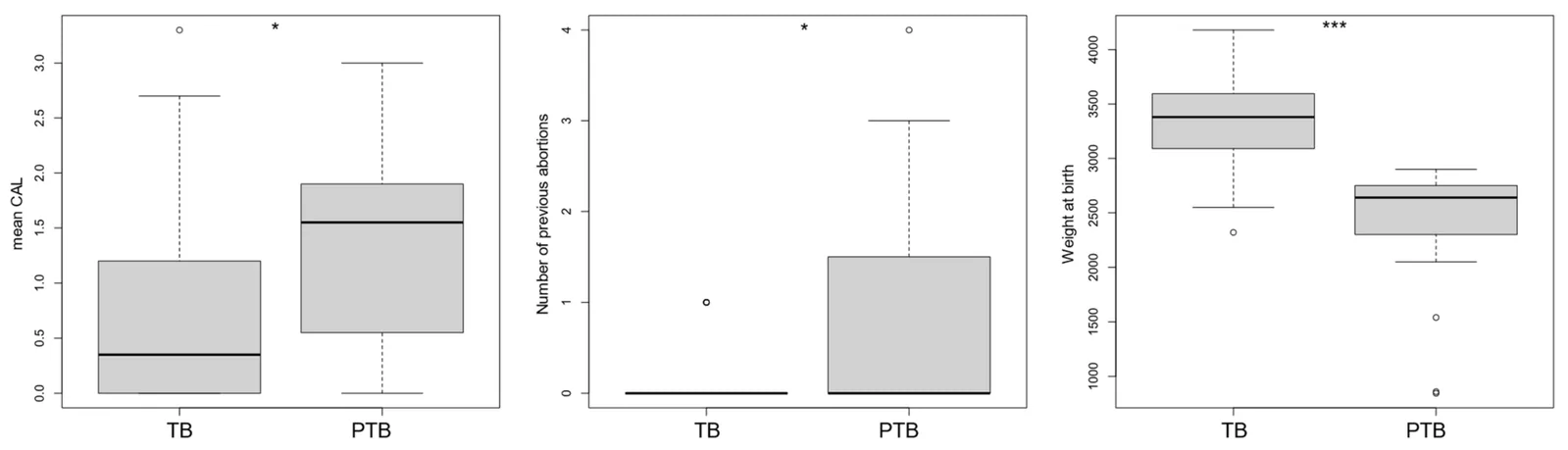
Impact of the clinical parameters and alpha diversity index on the risk factor, generalized linear model: mean CAL; number of previous abortions; weight of child. Note: TB = Term birth; PTB = Preterm birth; CAL = clinical attachment loss; * *p* < 0.05; *** *p* < 0.001; ° = outliers.

Finally, we compared the birth weight of babies of orally healthy women to women with gingivitis and initial periodontitis (Stage I), and also women with moderate and severe periodontitis (Stage II and III). Here, we found significantly higher birth weights in orally healthy women compared with the latter two pooled periodontal cohorts (*p* = 0.013 and *p* = 0.008, respectively; Table 4). Using a linear mixed model, we found a significant impact of the alpha diversity and mean CAL (*p* = 0.026), weight of child (*p* < 0.001) and number of previous aborts (*p* = 0.031) on occurrence of preterm birth (Figure 2).

### 3.2. 16S rRNA Sequencing Analysis

**Figure 3:**
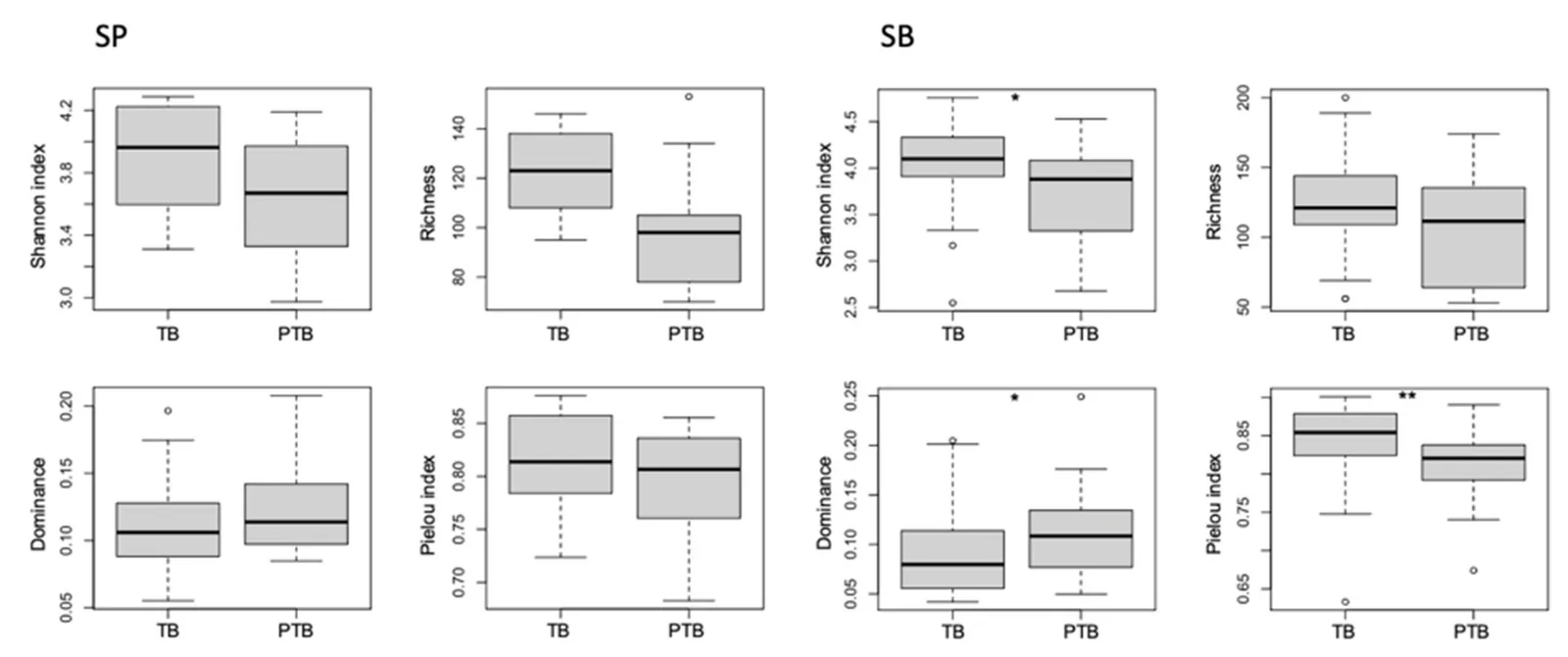
Impact of the groups on alpha-diversity, richness, evenness and dominance of supragingival (SP) and subgingival (SB) plaque. Note: SP = supragingival; SB = subgingival; TB = Term birth; PTB = Preterm birth; * *p* < 0.05; ** *p* < 0.01; ° = outliers.

PERMANOVA identified significant differences in microbiome composition between supra- (PTB *n* = 26; TB *n* = 31) and sub-gingival (PTB *n* = 11, TB *n* = 14) samples (*R*^2^ = 0.11, *p* = 0.001). In addition, the microbiome composition of supragingival and subgingival samples differed significantly between PTB and TB groups (*R*^2^ = 0.13 with *p* = 0.015 and *R*^2^= 0.08 with *p* = 0.002, respectively; Figure 3). The TB group was also found to have significantly higher α-diversity (*p* = 0.020), evenness (*p* = 0.006), and lower dominance (*p* = 0.019) than the PTB group in subgingival samples (Figure 3).

**Figure 4:**
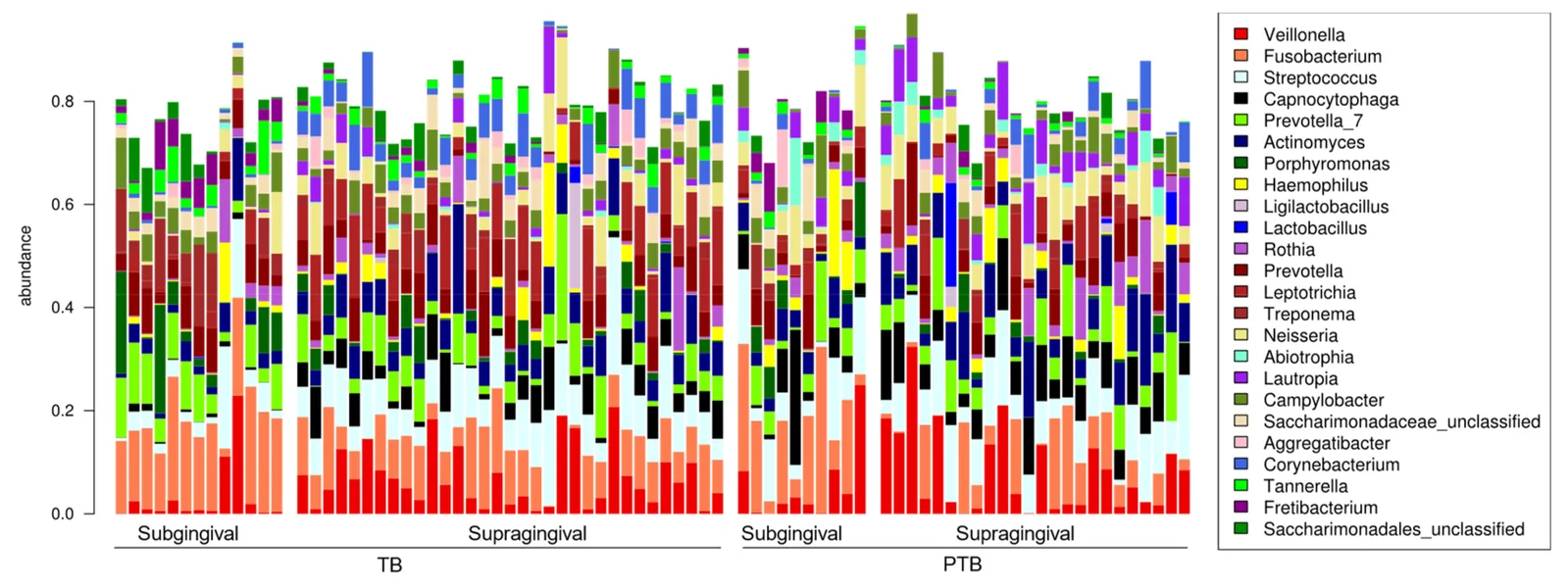
Barplot, abundance of supra- and subgingival samples for the preterm birth group (PTB) and term birth group (TB). Note: TB = Term birth; PTB = Preterm birth.

**Figure 5:**
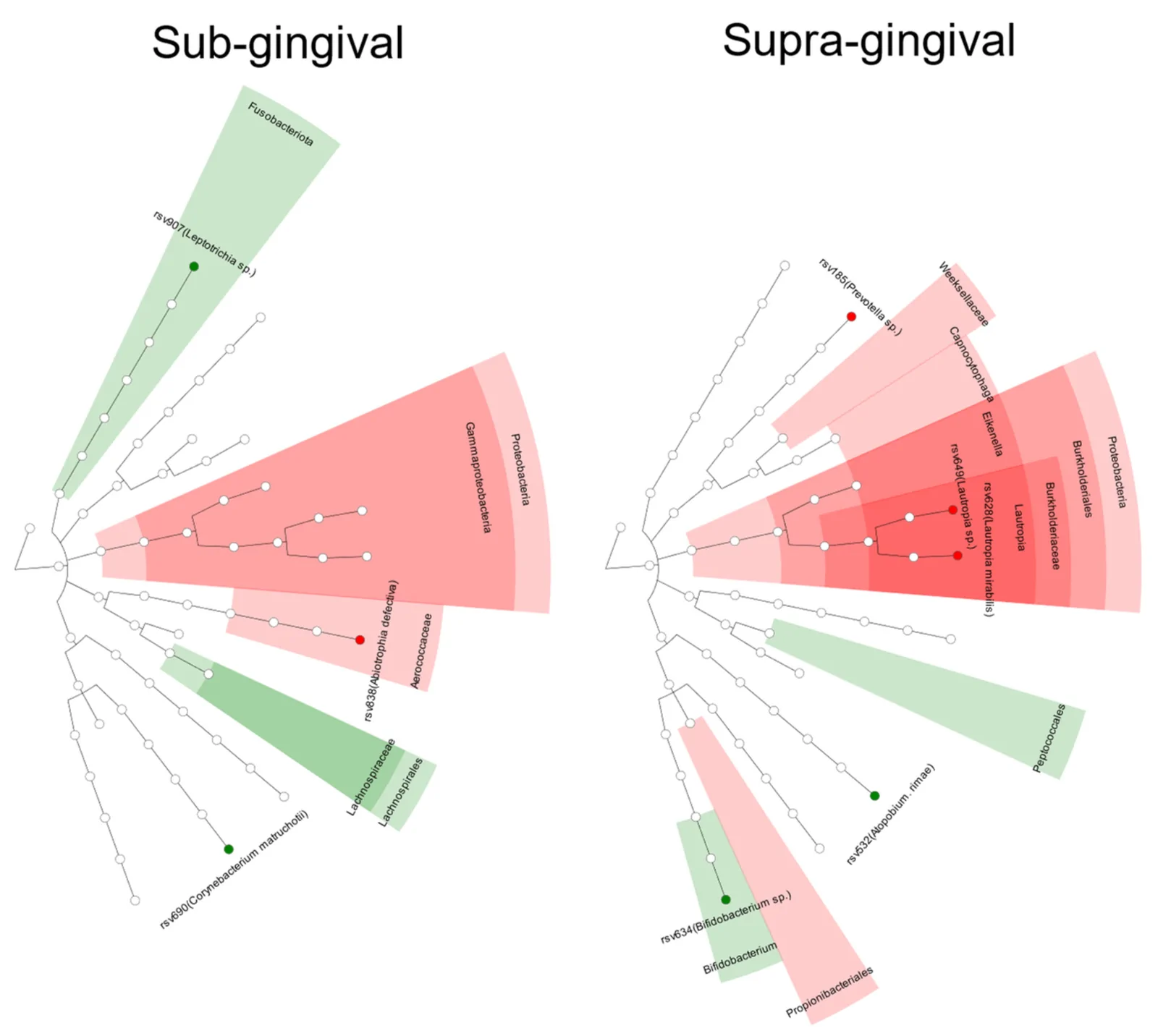
Differences in abundance of certain taxa between the groups (red = PTB, green = TB).

Our *DESeq2* analysis identified five RSVs that are differentially abundant in supragingival samples. While *Atopobium rimae* and *Bifidobacteria spp* were less abundant in the PTB group (-log_2_ fold changes of 7.90 and −7.75, respectively), *Lautropia spp*, *Lautropia mirabilis*, and *Prevotella spp* were instead more abundant (9.33, 3.89, and 9.27, respectively). In subgingival samples, three RSVs were differentially abundant in the PTB group: *Corynebacterium matruchotii* and *Leptotrichia spp* were less abundant (−1.99 and −5.19, respectively), while *Abiotrophia defectiva* was instead more abundant (3.91). An overall increase in proteobacteria was observed in both supragingival and subgingival samples in the PTB group compared with the TB group (Figure 4 and Figure 5).

## 4. Discussion

In this study, we investigated the oral inflammatory status and the compositions of the oral microbiome in women with PTB and compared them to women with TB. The *2017 World Workshop Classification of Periodontal Diseases* was used to set explicit and prospectively comparable disease definitions that were absent from previous studies, making them challenging to align and compare [39,53,54]. With a median age of 31 (IQR 27–34) years, the women in our cohort were below a maternal age of >35, which is considered a risk factor for preterm delivery [55]. The dental status represented by the DMFT index was similar in both groups. Notably, it was below representative values for the German population reported in *The Fifth German Oral Health Study* (DMS-V) and comparable to results in another study on pregnant women [56,57]. In a systematic review, PTB was not associated with higher DMFT [58]. While socioeconomic status has been reported to affect dental caries during pregnancy in our cohort [59], we did not observe a relationship between educational background and DMFT.

### 4.1. CAL, PPD, and PISA

The periodontal CAL and the relative number of PPD ≥ 4 mm were significantly higher in women with PTB. However, no significant associations were observed with GBI or BOP. Pathological periodontal pockets and tissue breakdown occur when immunological mechanisms fail to contain and resolve an existing gingival inflammation [60]. Consequently, dysbiosis, in combination with a florid immunological reaction, causes the increased breakdown of the periodontal connective tissue [61]. However, during pregnancy, increased periodontal pockets cannot result only from a periodontal breakdown but also from gingival enlargement [62], where increased pocket depths, but not increased CAL, are observed. Pregnant women are more prone to gingival inflammation, as the altered levels of sex steroids during pregnancy lead to higher vascularization of the gingiva and proliferation of fibroblasts [63,64]. In this cohort, the periodontal parameters CAL and PPD for women with PTB were aggravated, whereas BOP, PISA, and GBI were similar in women with TB and PTB. Both bleeding parameters were present in <30% of all measured sites, representing localized inflammation in both groups [41]. GBI and BOP are positively impacted by improved domestic oral hygiene procedures, which could arise due to a higher awareness of oral hygiene during pregnancy. Subgingival sites with >2 mm PPD are usually not accessible for domestic hygiene procedures [65,66]. Those inflamed areas need professional subgingival periodontal treatment. It can be assumed that the equal bleeding values of both groups are a result of a generally high awareness for domestic oral hygiene procedures. The number of sites with deep pockets and BOP was masked through the much higher number of sites with PPD <3– 4 mm. Therefore, no statistical difference in bleeding parameters could be demonstrated. Therefore, the higher relative number of pockets with PPD ≥ 4 mm in combination with periodontally induced clinical attachment loss points to the potential linkage of PTB with periodontitis and not gingivitis.

### 4.2. Birth Weight

Birth weight was significantly higher in periodontally healthy women than in women with gingivitis or periodontitis, which agrees with the findings of Offenbacher et al., where periodontal disease in postpartum women was a significant risk factor for low birth weight [67]. In general, low birth weight is associated with non-communicable diseases like cardiovascular or renal diseases. Moreover, low birth weight was related to increased morbidity compared to birth weight appropriate with the gestational age [68]. Therefore, while low birth weight results from early delivery in most cases, it can also occur at normal delivery timepoints. In addition to anthropomorphic aspects of the maternal body, birth weight is affected by maternal habits and factors such as transporting nutrients and toxins to the placenta [68]. It can be assumed that in the presence of increased severity of periodontal disease, bacterial toxins of periodontal origin might negatively affect fetal growth. Periodontal pathogen-specific antibodies in the umbilical cord’s blood have been reported as indirect evidence of periodontal pathogens present in the fetoplacental compartments [25].

### 4.3. Microbiome

A limited number of studies have used 16S rRNA gene sequencing technologies to investigate the oral microbiome of pregnant women [38]. To our best knowledge, this is the first study comparing the oral microbiome of women with PTB and TB. We explored changes in the supra- and sub-gingival microbiomes using 16S rRNA-gene sequencing of plaque samples. We found the α-diversity, dominance, and evenness of the microbiomes to be significantly affected by PTB. A significantly higher dominance and lower evenness within PTB microbiomes indicate a potential overrepresentation of certain bacteria and dysbiotic bacterial shift. Moreover, we found relationships between CAL, WOC, and the number of previous abortions and PTB, underscoring our other findings. Previous studies found high abundances of the phyla *Firmicutes*, *Bacteroidetes*, *Fusobacteria*, *Proteobacteria*, and *Spirochaetes* in pregnant women [69,70]. Here, *Leptotrichia* was more abundant in pregnant women with gingivitis compared to gingival health, while *Veillonella* was more abundant in women with gingivitis, and different operational taxonomic units (OTU) of the genus *Prevotella* were present in both groups [69]. 

We also found significant differences in the abundance of several RSVs between groups. *Atopobium rimae* has been associated with autoimmune diseases such as Systemic Lupus Erythematosus [71] as well as chronic tonsillitis, infection-related glomerulonephritis, and splenic abscesses [72,73,74] and with IL−1beta and IL8 in GCF [75]. Here, it was less abundant in the supragingival samples of our PTB group. *Bifidobacteria spp* associated with periodontal health [76] as well as caries [77] were also less abundant (−log_2_ fold changes of −7.90 and −7.75) in supragingival plaque of PTB. *Lautropia spp* and *Lautropia mirabilis* were associated with periodontal health [78]. *Prevotella spp* were also more abundant (−log_2_ fold changes of 9.33, 3.89, and 9.27) in the PTB group. *Prevotella* belongs to the phylum Bacteroidetes, a common periodontal pathogen of the orange complex [79], and is associated with bacterial vaginosis [80]. *Prevotella* and other oral species may impair neutrophil leukocyte function and produce collagenases and fibrinolysins, which may collectively contribute to the induction of preterm birth [81]. A connection between *Prevotella* and preterm birth has also been reported in a study investigating the vaginal microbiome and metabolome of women with preterm membrane rupture, which found *Prevotella* to be among the most abundant species within the preterm membrane rupture group [82].

Moreover, in agreement with the findings of Miranda-Rius, we found a higher abundance of proteobacteria but not actinobacteria within the supragingival samples of the PTB group [83]. Proteobacteria are generally associated with periodontal health, particularly *L. mirabilis*, which was identified as part of the core microbiome [78,84]. Therefore, the overabundance of proteobacteria illustrates that the supragingival microbiomes in PTB women do not undergo a substantial pathogenic shift and still display a partially healthy profile. The pathogenic shift occurs in the periodontal pockets and is driven by the more pronounced subgingival inflammatory environment [85].

In subgingival samples, three RSVs were differentially abundant in PTB and TB groups: (1) *Corynebacterium matruchotii*, which is known to form corncobs in human plaque and is part of the oral core-microbiome [86]; (2) *Leptotrichia spp,* which is also part of the core oral microbiome and, when overabundant, associated to halitosis and oral leukoplakia [87,88] was less abundant (−log2 fold changes of −1.99 and −5.19, respectively); and (3) *Abiotrophia defectiva* was more abundant (−log2 fold change = 3.91). An overall increase in proteobacteria was observed in both supragingival and subgingival samples from the PTB group.

The microbial profile of placentas based on 16S rRNA sequencing was recently reported by Miranda-Rius et al. (2021). In this study, three groups were compared: (1) women with adverse pregnancy outcomes and periodontitis; (2) women with adverse pregnancy outcomes without periodontitis; and (3) periodontally healthy women with TB. The diversity of the placental microbiome was found to differ significantly between the groups, where periodontitis was a more discriminant variable than adverse pregnancy outcomes, and a principal coordinates analysis (PCoA) found significant differences in β-diversity (*p* < 0.001). Moreover, at the phylum level, proteobacteria and actinobacteria were associated with the presence of periodontitis in combination with adverse pregnancy outcomes. In addition, at the family level, aerococcaceae were associated with periodontitis but not adverse pregnancy outcomes [83]. This observation is consistent with our findings, where aerococcaceae *A. defectiva* was associated with PTB in women with periodontitis. *Abiotrophia spp* is an apparent potent inducer of proinflammatory cytokines [89], and *A. defectiva* is reportedly associated with endocarditis [90], indicating it is a pathogen with high systemic inflammation-inducing activity.

The major periodontal pathogen *Porphyromonas gingivalis*—a periodontal keystone pathogen [16]—was found in the amniotic fluid and the oral cavity of a subgroup of women with PTB and periodontitis [91]. In our study, there was no significant difference in the abundance of *P. gingivalis* between groups (−log_2_ fold change = 1.23, *p* = 0.999). Similarly, *P. gingivalis*, *T. forsythensis*, *T. denticola*, and *F. nucleatum* abundance did not differ significantly in women with PTB and TB in a study using Checkerboard DNA–DNA hybridization [92]. Since *P. gingivalis* is a widespread periodontal pathogen, its equal abundance in both our study groups is to be expected. Notably, it is a potent inducer of dysbiotic conditions and, with its subversive abilities regarding immunological defense mechanisms, a potent inducer of pathogenic conditions [93]. As subgingival dysbiosis progresses, the relative quantity of periodontal pathogens generally increases in periodontal pockets, and thus the chance of systemic dissemination increases [94]. The ulceration of the gingival epithelium and hyperpermeability of blood vessels favors the invasion of bacterial products, inflammatory mediators, and microorganisms into the bloodstream, elevating the risk of effects in distant body sites [25]. Therefore, systemic dissemination of *P. gingivalis* might occur in its presence, with even domestic mechanical plaque removal associated with systemic bacteriemia [2,95].

In summary, our sequencing results on the oral sub- and supra-gingival microbiome indicate a possible association of the microbial composition and specific genera and species with the occurrence of PTB. The exact role of specific species, their interactions, and the impact of their metabolic products on PTB represent urgent areas for future research. Future studies with larger numbers of participants should evaluate differences in the oral and placental microbiome, using not only 16srRNA but full metagenome sequencing and proteome and metabolome analyses.

Significant limitations of our study were the size of its cohort and that we were not able to include the full calculated sample size. Hence, some *p*-values need to be interpreted with caution. Moreover, it was not possible to conduct all examinations on all women. These factors were partly due to participants who had recently given birth being in a stressful situation in an unfamiliar clinical environment, where psychological strain and physiological burden negatively impacted their willingness to participate in this study. The long hospitalization period of some women prior to examination may have reduced oral hygiene, even though gingival inflammatory conditions were not elevated in our cohort. This factor might not have affected clinical attachment loss but could affect the pocket depth. The number of decayed teeth was assessed only clinically and without radiographs due the hospitalization of the patients. Nevertheless, a strength of our study is the detailed periodontal examination with measurements on six sites per tooth, including clinical attachment loss and the use of the classification of periodontal diseases recommended in 2017. These factors will improve the comparability of our findings in future studies. In addition, the use of 16S rRNA sequencing advanced our understanding of oral microbiome composition and increased the feasibility of future comparisons between the oral and placental microbiome. Our findings highlight the importance of professional oral care before and during pregnancy, especially in diagnosing and treating periodontal disease.

## 5. Conclusions

Women with PTB had higher clinical attachment loss and a significantly higher percentage of sites with PPD ≥ 4 mm. Birth weight was significantly higher in orally healthy women than in those with gingivitis or periodontitis. The composition of the supra- and sub-gingival microbiome was significantly different between the groups, including changes in abundance of some supra- and sub-gingival RSVs in women with PTB compared to women with TB. Further studies with a broader patient collective are required to confirm and expand our findings.

## Figures and Tables

**Table 1:** Demographic and pregnancy outcome data: median with interquartile range (IQR).

	*n*	Preterm Birth *n* = 33	Term Birth *n* = 44	*p*-Value
Age (years)	77	33 (range: 21–44)	29.5 (range19–41)	0.0881
smoker (*n*. %)	72	1 (3.23%)	7 (17.07%)	0.0641
Delivery week	77	35.14 (34.00–36.14)	39.64 (38.75–40.71)	<0.0001
Delivery mode:	77			
spontan (*n*. %)		8 (24.2%)	22 (50.0%)	0.0018 *
primary sectio caesarea. *n*		2 (6.0%)	9 (20.4%)	
secondary sectio caesarea. *n*		17 (51.5%)	6 (13.6%)	
VE. median. *n*		6 (18.18%)	7 (15.9%)	
Birth weight	73	2620 (2285.00–2787.50)	3375 (3090.00–3615.00)	<0.001
Birth weight < 2500 g (*n*. %)		13 (41.94%)	1 (2.4%)	
Gestational DM (*n*. %)	71	3 (9.68%)	3 (7.5%)	0.7436
Previous miscarriage	76	0 (range 0–4)	0 (range 0–1)	0.0326

*n* with percentage. *p*-value by Mann–Whitney U Test or Chi-Square Test; VE = vacuum extraction; DM = Diabetes mellitus. *p* < 0.05.

**Table 2:** Oral clinical data and labor data. Median with IQR.

	*n*	Preterm Birth *n* = 33	Term Birth *n* = 44	*p*-Value
BOP (%)	70	18.2 (13.1–29.8)	27.1 (14.8–44.8)	0.0811
PISA (%)	70	241.40 (151.67–365.76)	316.82 (146.91–506.26)	0.2058
GBI (%)	72	14.15 (10.48–19.98)	19.9 (6.73–36.68)	0.5718
DMF(T). median	74	9.0 (5.5–11.5)	5.0 (2.0–10.5)	0.0911
PPD (mm)	70	2.1 (2.0–2.3)	2.1 (2.0–2.2)	0.6041
CAL (mm)	70	1.4 (0.4–1.9)	0.4 (0.0–1.2)	0.0128
*n* PPD ≥ 4 mm	70	5 (2–10)	2 (0–6)	0.0579
Rel. PPD ≥ 4 mm	70	2.98 (1.19–5.95)	1.19 (0–3.85)	0.047
Number of lost teeth	73	0 (range 0–5)	0 (range 0–8)	0.7609

*p*-value by Mann–Whitney U Test; BOP = bleeding on probing, PISA = Periodontal Inflamed surface area; GBI = Gingival bleeding index; DMF(T) = decayed, missing, filled (teeth); PPD = probing pocket depth; CAL = clinical attachment loss.

**Table 3:** Periodontal diagnosis according to birth outcome.

*n* = 70	Preterm Birth (*n*. %)	Term Birth (*n*. %)	Age (Median, Range)	Smoker
Periodontal health	1 (3.2%)	6 (15.4%)	28 (23–40)	1 (16.7%)
Gingivitis	12 (28.7%)	20 (51.3%)	30 (19–39)	3 (9.7%)
Periodontitis				
Gen. Stage I	11 (15.5%)	8 (20.5%)	32 (25–44)	2 (11.1%)
Gen. Stage II	6 (19.4%)	4 (10.3%)	31.5 (21–41)	1 (10%)
Gen. Stage III	1 (3.23%)	1 (2.6%)	29.5 (25–34)	0

**Table 4:** Birth outcome parameters and labor data according to periodontal diagnosis. Explorative *p*-values by Kruskal–Wallis Test without Bonferroni correction.

	Periodontal Diagnosis Groups				
	Periodontal Health (*n* = 7)	Gingivitis and Periodontitis Stage I (*n* = 51)	Periodontitis (*n* = 12)	*p*-Value (Periodontal Health vs. Periodontitis)	*p*-Value (Periodontal Health vs. Gingivitis)
Birth weight	3615 (± 407.59)	2924.27 (± 721.9)	2710 (± 684.67)	0.0127	0.0078
Delivery week	39.02 (± 2.01)	37.21 (± 3.61)	36.93 (± 2.63)	0.0688	0.1973

## Data Availability

The data presented in this study are available on request from the corresponding author.

## Supplementary Materials

The following supporting information can be downloaded at: https://www.mdpi.com/article/10.3390/jcm11237183/s1, Table S1: STROBE Statement—Checklist of items that should be included in reports of case-control studies. 
